# Nursing Interventions to Facilitate the Grieving Process after Perinatal Death: A Systematic Review

**DOI:** 10.3390/ijerph18115587

**Published:** 2021-05-24

**Authors:** Alba Fernández-Férez, Maria Isabel Ventura-Miranda, Marcos Camacho-Ávila, Antonio Fernández-Caballero, José Granero-Molina, Isabel María Fernández-Medina, María del Mar Requena-Mullor

**Affiliations:** 1 Faculty of Health Sciences, University of Granada, Distrito Sanitario Almería, 04009 Almería, Spain; albafferez@correo.ugr.es; 2 Department of Nursing, Physiotherapy and Medicine, University of Almería, 04120 Almería, Spain; jgranero@ual.es (J.G.-M.); isabel_medina@ual.es (I.M.F.-M.); mrm047@ual.es (M.d.M.R.-M.); 3 Obstetrics Service, Hospital La Inmaculada, 04600 Huércal-Overa, Spain; cam382@inlumine.ual.es; 4 Faculty of Nursing, Univesity of Cádiz, 11207 Algeciras, Spain; antonio.fernandez@uca.es; 5 Gynecology, Obstetrics and Delivery Service of the Hospital Punta Europa, 11207 Algeciras, Spain; 6 Faculty of Health Sciences, Universidad Autónoma de Chile, Santiago 7500000, Chile

**Keywords:** perinatal death, perinatal loss, perinatal grief, nursing, midwifery

## Abstract

Perinatal death is the death of a baby that occurs between the 22nd week of pregnancy (or when the baby weighs more than 500 g) and 7 days after birth. After perinatal death, parents experience the process of perinatal grief. Midwives and nurses can develop interventions to improve the perinatal grief process. The aim of this review was to determine the efficacy of nursing interventions to facilitate the process of grief as a result of perinatal death. A systematic review of the literature was carried out. Studies that met the selection criteria underwent a quality assessment using the Joanna Briggs Institute critical appraisal tool. Four articles were selected out of the 640 found. Two are quasi-experimental studies, and two are randomized controlled clinical studies. The interventions that were analyzed positively improve psychological self-concept and role functions, as well as mutual commitment, depression, post-traumatic stress and symptoms of grief. These interventions are effective if they are carried out both before perinatal loss and after it has occurred. The support of health professionals for affected parents, their participation in the loss, expressing feelings and emotions, using distraction methods, group sessions, social support, physical activity, and family education are some of the effective interventions.

## 1. Introduction

Perinatal death (PD) is the death of a baby that occurs between the 22nd week of pregnancy (or more than 500 g of weight) and seven days after birth [1]. PD is a global health problem, and although it has been reduced, 2.6 million perinatal deaths occurred in the world in 2017 [2]. In Europe, there were between 4 and 6 deaths per 1000 births [3]. In Spain, perinatal deaths have decreased from 20 deaths per 1000 births in 1975 to 4.37 deaths per 1000 births in 2019 [4]. PD can occur as a result of maternal conditions [5] such as high blood pressure [6], anemia [7], diabetes, bacterial infections; fetal conditions such as congenital anomalies, intrauterine growth retardation, or multiple pregnancy; placental conditions such as premature detachment of the placenta or bleeding; uterine conditions such as uterine malformation; delivery conditions such as asphyxia during delivery hypoxia [8], umbilical cord prolapse [9], or complications of caesarean section [10] and other unclassifiable or unclear conditions [11,12].

After PD, parents experience the perinatal grief process [13], which affects them on biopsychosocial and spiritual levels [14]. Thus, parents may experience depression, anxiety, post-traumatic stress disorder [13], eating and sleeping disorders [15], isolation and loss of faith [16], among other symptoms. The incidence of PD, as well as its important consequences, suggests the need to train nurses in this area [17]. Such training is essential in order to offer the highest possible quality of care based on the needs of the affected fathers and mothers [18]. Furthermore, good professional care based on empathy and emotional support has been shown to help families in the grieving process [19]. Professionals can also suggest practical measures to help the bereaved to cope with and overcome grief, such as holding the baby, and taking a photograph of the moment. Support groups are also effective in dealing with a loss of this type [20].

Nurses and midwives often perform interventions to help parents who have suffered a perinatal death, in fact, nurses are one of the main sources of support. However, the important work of nurses in these situations is hampered by the lack of recognized strategies that can be implemented to assist parents and family members in the grieving process [21]. Additionally, are these interventions effective in helping fathers and mothers who have suffered a perinatal loss to deal with or resolve grief? Although there was a recent review of the literature on the efficacy of interventions on grief after an abortion [22], perinatal grief (between 22 weeks and one week of life) has not been well studied. A specific review on this topic (PD) published in 2013 did not find studies of interventions specifically focused on perinatal bereavement [23]. Therefore, eight years later, a systematic review can be justified; the aim of this review was to analyze the available evidence on the efficacy of nursing interventions to facilitate the process of grief as a result of perinatal death.

## 2. Materials and Methods 

### 2.1. Design 

We conducted a systematic review of studies of interventions by following the Cochrane Handbooks [24] and the recommendations of the Preferred Reporting Items for Systematic Reviews and Meta-Analyses (PRISMA) guideline, [25].

### 2.2. Search Methods

A literature search was conducted using four electronic data bases (PubMed, Scopus, Web of Science and Cochrane Library) between January and June 2020. The PICO question was used as a search strategy to identify possible studies, using a combination of natural and structured language, and Medical Subject Headings (MeSH).

The keywords were systematically combined in order to conduct the literature search: “perinatal bereavement” AND “nursing care”; “perinatal death” AND “nursing”; “perinatal loss” AND “nurse”; “perinatal grief” AND “nurse”. The databases were queried using titles, abstracts, and the keywords. 

In addition to searching the cited databases for studies, specific searches were made in a number of specialized journals such as *Advanced Neonatal Care*, and *BMC Pregnancy and Childbirth*. The reference lists of included articles were also reviewed for additional sources.

The document selection was carried out independently by two researchers, and disagreements between them were settled by consensus. First, previously unidentified duplicates were eliminated. Then the titles and abstracts of the remaining articles were screened to identify eligible papers reviewed in full text.

### 2.3. Selection Criteria

The criteria for inclusion of studies in this review were: (1) studies published in the last 5 years; (2) studies published in English or Spanish; (3) quasi-experimental studies or randomized clinical trials (RCTs).

The criteria for exclusion were: (1) studies on interventions in women who are less than 14 weeks pregnant; (2) studies that do not describe specific interventions in a group of women. 

### 2.4. Search Outcomes

In the identification phase, the search of the data bases yielded 590 articles and 50 articles from other previously mentioned sources, for a total of 640 articles. We checked how many of them were duplicates (270), which resulted in a total of 370 articles.

**Figure 1:**
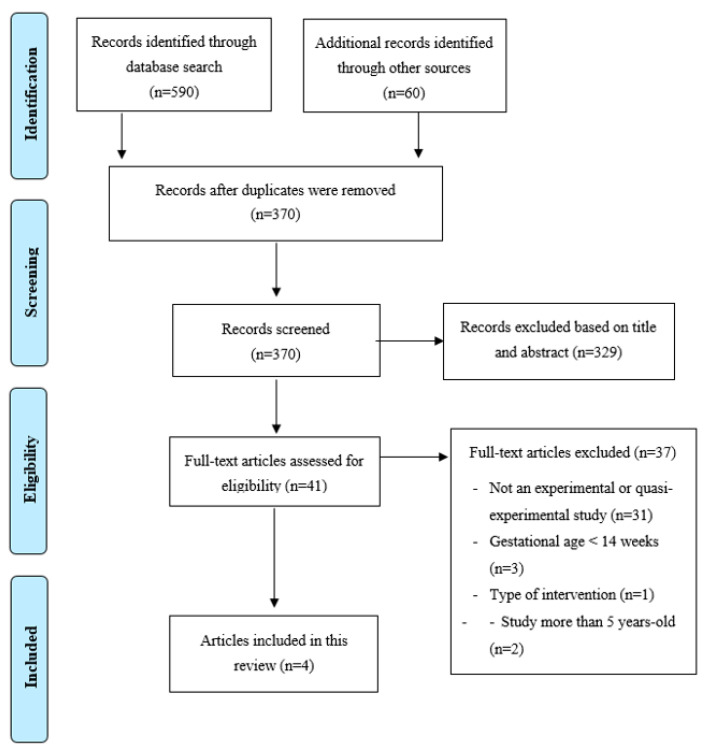
PRISMA study flowchart.

In the screening phase, after reading the title, 329 studies were discarded, with 41 articles remaining after the eligibility phase. After reading the abstract, 31 were eliminated, and another 6 were discarded for other reasons as shown in Figure 1. There were 4 articles left in the inclusion phase (Figure 1).

### 2.5. Quality Appraisal

The methodological quality of the studies was assessed using the Joanna Briggs Institute (JBI) critical appraisal tool [26]. There were 9 questions to guide the evaluation of quasi-experimental studies and 13 for the evaluation of RCTs. The questions were quantified using scores from 0 to 1. One point is given if the answer is “YES” and zero points if the answer is “NO”, “not clear” or “not applicable.” (Table 1 and Table 2).

### 2.6. Risk of Bias 

**Figure 2:**
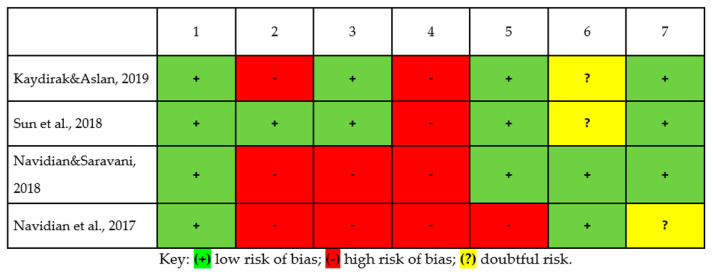
Risk of bias summary. Criterion number: 1, Random sequence generation (selection bias); 2: Allocation concealment (selection bias); 3: Blinding of participants and personnel (performance bias): 4: Blinding of outcome assessment (detection bias); 5: Incomplete outcome data (attrition bias); 6: Selective reporting of results (reporting bias); 7: Other bias (conflict of interests).

We applied the Cochrane Collaboration tool [24] to assess the risk of bias in the studies, rating each criterion as having a high, unclear, or low risk of bias (Figure 2). All four studies were low risk in relation to random sequence generation. One study reported low risk for allocation concealment bias, and the rest were high risk. Two of the studies provided sufficient information on the blinding of the participants, and their risk of performance bias was rated as low, while the other two studies did not provide sufficient information and were therefore classified as high risk. All four studies had a high risk of detection bias, as they did not clearly describe the methods used to blindly assess the outcome measures. A high risk of attrition bias was observed in one of the studies, with the rest of the studies presenting a low risk. Regarding selective reporting bias, two of the studies had borderline risk and the other two had low risk. Finally, one study showed an unlikely risk of conflict of interest.

### 2.7. Data Abstraction and Synthesis 

Two independent reviewers (A.F.-F., M.I.V.-M.) independently selected and extracted information from the four studies included in this review. Following the Cochrane Handbook, we created a template with the following titles for reviewers to independently extract the information: first author’s name, year of publication, study location, study design, sample size, duration of intervention, characteristics of the participants (gestational age and age of women), characteristics of the intervention, outcome measures, and main findings. The reviewers used this template to extract data from all four studies. Then a third experienced reviewer, M.M.R.-M., verified the extracted data. A summary of the extracted data can be seen in Table 3. We carried out a narrative synthesis of the findings based on the characteristics of the interventions, the variables studied, and the reported findings.

## 3. Results

### 3.1. Narrative Summary

This systematic review included four studies out of a total of 640. Of the four studies chosen, two of them were randomized controlled clinical trials (RCTs), and two were quasi-experimental intervention studies. The two RCTs studied women who were pregnant and required termination of pregnancy. The two quasi-experimental studies examined women who had experienced perinatal or fetal death prior to the intervention. Table 3 shows the main characteristics of the included studies. 

Navidian et al. [27] determined the effect of behavior-based cognitive counselling on the severity of bereavement symptoms in mothers after fetal death. They conducted a quasi-experimental study with a sample of 100 pregnant women. 

In addition, Navidian et al. [28] determined the effect of grief counselling on the severity of symptoms of post-traumatic stress disorder in mothers after fetal death in a quasi-experimental study with a sample of 100 pregnant women. 

Sun et al. [29] analyzed whether a family support program could improve family support and alleviate depression and post-traumatic stress symptoms in pregnant women with fetal abnormalities. To do this, they performed a randomized controlled trial with a sample of 124 pregnant women. 

Kaydiraket al. [30] evaluated the effectiveness of a nursing support program for the medical termination of pregnancy, according to the Roy adaptation model (physiological adaptation, self-concept, function, and mutual dependence). In order to do this, they conducted a randomized controlled trial with a sample of 77 pregnant women.

### 3.2. Intervention Details

The duration and frequency of the interventions in the studies varied with regard to the interviews conducted with the experimental group and the control group. This is the case of one of the studies that conducted five interviews with the experimental group and two with the control group [30]. Another study carried out the intervention over a 6-week period and then did follow ups for 15 to 20 min by telephone or WeChat (platform created) 2 weeks after the birth [29], and the other two studies reviewed carried out the intervention in 60 min sessions over two weeks [27,28].

All of the studies performed the interventions at an individual level except one, which did so at a group level [29]. The interventions were carried out by nurses in one of the studies [30], and in another, by a multidisciplinary team (a psychologist, two obstetric doctors, a researcher and two nurses) [29]. The other two remaining studies did not specify who performed the interventions [27,28].

### 3.3. Characteristics of Participants

The participants were pregnant women with a gestational age of more than 20 weeks [30], women who had experienced still birth (at least 7–10 days after loss) and a gestational age of more than 22 weeks [27,28], and women with a history of fetal death (1 to 3 perinatal losses) with a gestational age between 28–29 weeks and full term (28), and were at least 18 years old [27,28,29,30].

### 3.4. Intervention Outcomes

The interventions analyzed are used to improve the level of anxiety, post-traumatic stress, depression, and the symptoms of grief. These interventions are effective if they are performed both before perinatal loss and after it has occurred. In all four studies, the results were significantly more positive in the intervention group; however, in one, the psychological, self-concept and role functions, as well as mutual commitment improved significantly in the experimental group except in terms of anxiety and nausea and vomiting [30]. In two of the studies, counselling given to mothers reduced grief symptoms [27,28]. In another study, family APGAR scores and depression and post-traumatic stress scores were significantly better in the intervention group [29].

## 4. Discussion

In this review, we systematically examined the efficacy of interventions to help grieving as a result of perinatal death. Four studies met the selection criteria and were subjected to a critical quality analysis. Two of them report interventions performed before perinatal death [29,30], and two studies examine interventions performed after perinatal death had occurred [27,28]. The interventions were found to vary in duration, frequency, and content, making comparison between studies difficult [24].

Interventions performed prior to perinatal death show that support based on Roy’s adaptation model (the process and outcome by which people with thoughts and feelings, individually or in groups, use conscious awareness and choose to create human and environmental integration) and social support both help with coping and reduce anxiety in women [30]. The information that parents receive about what they are going to experience is important [31]. Parents must be prepared and informed about the process [29].

In the interventions carried out after perinatal loss, it was concluded that psychoeducation, psychotherapies, physical activity, and group sessions improved depression and sleep [27,28]. This is consistent with the studies by Beddoe et al. [32], which show that physical activity reduces stress and anxiety, and Nikčević et al. [33], who found that psychological support improved stress and depression symptoms. Similarly, Shaohua and Shorey concluded that psychosocial interventions are effective in improving depression, anxiety, and grief amongst parents with perinatal loss [34], and a study by Fuentetaja and Villaverde suggests that group sessions potentialize the individual capacities of each person [35].

Not all the studies analyzed involved the family or the spouse in the interventions. Other studies suggest that some relatives have insufficient knowledge or skills and resort to avoidance of the topic, which leads to increased anxiety [36]. Educating women, spouses and extended family about PD and grief is a good way to improve the grieving process [37].

Health education could be useful in places where the education level of the population is lower [38]. In other, more technologically advanced contexts, where this knowledge can be accessed more easily, technological tools such as online yoga [39] could be used. Kaydirak and Aslan used telephone calls to contact women and provide follow up [30]. However, Navidian and Saravanide showed that face-to-face (patient–therapist) psychotherapy interventions are more effective for grief symptoms [27].

Other studies mention the importance of the physical conditions of hospital rooms [18] and of women having a voice in decision-making (some women are afraid of not being able to do so) [40].

Despite the number of interventions that demonstrate an improvement in the grieving process, none of the studies that we analyzed mention other ways to facilitate grief, for example, photographing the baby, keeping a memory box or holding the baby after delivery. However, there are other types of qualitative research studies that address these issues. There are different opinions regarding these coping measures, some parents state that seeing the dead baby is an opportunity to say “goodbye” and fix it in their memory [41]. These parents suffer less post-traumatic stress than those who have not seen their baby [42]. On the other hand, the shock that this causes and the decision not to see the baby in order to cut the attachment may avoid greater suffering [41] as this can cause considerable anxiety [43]. However, no study has been found that has conducted experimental research to find the efficacy of these coping measures, so we can only be guided by the opinions of parents through interviews.

Inevitably, this analysis raises many questions such as: What do mothers and parents need? How do they prefer to cope with their loss? Do they want to hold the baby? How much privacy should they be given? Do we care for them on the postnatal ward with other mothers and healthy babies?

These are questions that any professional would ask, and they are difficult to answer without input from parents. It is essential to create a link with parents and facilitate communication between health professionals and the patient in order to know their needs [44]. For health professionals, this is not so simple; nurses and midwives report not knowing how to create memories of the baby for the parents or not having sufficient skills in grief counselling [45]. The coping strategies mentioned above are rarely used in hospitals; in one study, 20% of participating midwives had never offered mementos of the baby, and only 20% of them had suggested taking a photo of the baby [46]. Studies have highlighted the importance of nurses and midwives being prepared [47] and trained [48] to implement interventions for perinatal grief [49] since this group is best positioned to be “constant caregivers” [50].

### Limitations

Study limitations include the intervention by Navidian and Saravani [27], which was performed within the first weeks after the loss of the baby (7–10 days had passed since the baby’s death). This hinders the accuracy of the data since symptoms are stronger initially and may improve not only with the intervention but also with the passage of time and the assimilation of loss [51]. The two interventions by Navidian [27,28] are two different studies that use the same participants and the same interventions, but each one uses a different outcome assessment tool, the grief scale [27] and post-traumatic stress [28].

This systematic review has several limitations. Firstly, given the scarcity of intervention studies, including a wider variety of quantitative and qualitative designs would have provided more information, and more global, but less precise, conclusions could have been drawn.

Secondly, only studies in Spanish and English were recovered. Including other languages (Chinese, French, Portuguese) could have provided additional studies. 

Finally, we found studies in which women with a wide range of gestational ages were subject to interventions. Some women met the criterion of at least 14 weeks, others lost their baby at a higher gestational age, and others did so earlier. However, this wide range was considered too broad, with the interventions and levels of anxiety and grief varying greatly from week to week. Including these studies could have resulted in a higher number of articles and different viewpoints.

## 5. Conclusions

The interventions that were helpful to parents after PD were the support of health professionals for those affected, health education to understand the process and allow them to take part in the loss, the expression of feelings and emotions, distraction methods, group sessions, social support, physical activity and family education. These activities improve anxiety, feelings of grief, sleep, and self-confidence.

These support interventions for families experiencing perinatal loss are effective if they are carried out both before (if foreseeable) and after the death of the baby.

The interventions are mainly focused on the woman whereas the fathers remain in the background, even though they also feel the loss. Studies are needed that include fathers in nursing activities and interventions.

To address perinatal death in the right way, health professionals must feel safe and well trained. It is necessary to train nurses to face these circumstances with confidence, compassion and confidence.

## Figures and Tables

**Table 1:** Analysis of quasi-experimental studies with the JBI Checklist [26].

	Navidian & Saravani (2018) [27]	Navidian et al. (2017) [28]
1. Is it clear in the study what is the ‘cause’ and what is the ‘effect’	YES	YES
2. Were the participants included in any comparisons similar	YES	YES
3. Were the participants included in any comparisons receiving similar treatment/care, other than the exposure or intervention of interest?	YES	YES
4. Was there a control group?	YES	YES
5. Were there multiple measurements of the outcome both pre and post the intervention/exposure?	YES	YES
6. Was follow up complete and if not, were differences between groups in terms of their follow up adequately described and analyzed?	NA ^1^	NA ^1^
7. Were the outcomes of participants included in any comparisons measured in the same way?	YES	YES
8. Were outcomes measured in a reliable way?	YES	YES
9. Was appropriate statistical analysis used?	YES	YES
TOTAL SCORES	8/9	8/9

^1^ NA: Not applicable.

**Table 2:** Analysis of the ECAS studies with the JBI Checklist [26].

	Sun et al. (2018) [29]	Kaydirak & Aslan (2019) [30]
1. Was true randomization used for assignment of participants to treatment groups?	YES	YES
2. Was allocation to treatment groups concealed?	YES	YES
3. Were treatment groups similar at the baseline?	YES	YES
4. Were participants blind to treatment assignment?	YES	YES
5. Were those delivering treatment blind to treatment assignment?	NO	NO
6. Were outcomes assessors blind to treatment assignment?	NO	NO
7. Were treatment groups treated identically other than the intervention of interest?	YES	YES
8. Was follow up complete and if not, were differences between groups in terms of their follow up adequately described and analyzed?	YES	YES
9. Were participants analyzed in the groups to which they were randomized?	YES	YES
10. Were outcomes measured in the same way for treatment groups?	YES	YES
11. Were outcomes measured in a reliable way?	YES	YES
12. Was appropriate statistical analysis used	YES	YES
13. Was the trial design appropriate, and any deviations from the standard RCT design (individual randomization, parallel groups) accounted for in the conduct and analysis of the trial?	YES	YES
TOTAL SCORES	11/13	11/13

**Table 3:** Summary of studies included in the review.

Author, (Year), Location	1. Design 2. Sample Size 3. Duration of the Intervention	Participants 1. Gestational Age/Fetal Death 2. Age of the Women	Intervention	Outcome Measures
Kaydirak et al., (2019), Turquía	1. Randomised Controlled Trial 2. 77 3. Pre/post	1. >20 weeks gestation 2. ≥18 years old	Nurses implanted the TNSP ^1^ based on the Roy model ^2^	Positively affects psychological, self-concept and role functions, and mutual commitment
Sun et al., (2018), China	1. Randomized Controlled Trial 2. 124 3. Pre/post	1. Women with a history of still birth (1 to 3 perinatal losses) 2. ≥18 years old	Multidisciplinary team (a psychologist, two obstetric doctors, a researcher and two nurses) developed a family support programme, including an information support package (educational brochure), family support education, postpartum counselling, and real time communication via WeChat	Family APGAR ^3^ scores and depression and post traumatic stress scores were significantly better in the intervention group
Navidian et al. (2018), Irán.	1. Quasi-experimental 2. 100 3. Pre/Post/ 2 weeks	1. 7–10 days after the loss 2. 18 years old	4 sessions on the history of grief, experience, stages and cycle of grief, exposure of feelings and thoughts, cognitive restructuring, meaning of loss and coping techniques and methods, among other therapies.	Counselling reduced grief symptoms.
Navidian et al., (2017), Irán	1. Quasi-experimental 2. 100 3. Pre/post 2 weeks	1. 7–10 days since the loss 2. 18 years old	4 sessions on the history of grief, experience, stages and cycle of grief, exposure of feelings and thoughts, cognitive restructuring, meaning of loss and coping techniques and methods, among other therapies.	Post traumatic stress score was reduced in the group that received the intervention

^1^ TNSP (Nursing support program on the termination of pregnancy). ^2^ Roy model (physiological adaptation, self-concept, function and mutual dependence). Family APGAR ^3^ (is an instrument that shows how family members perceive the level of functioning of the family unit in a global way).
